# Increased Survival of *Lactococcus lactis* Strains Subjected to Freeze-Drying after Cultivation in an Acid Medium: Involvement of Membrane Fluidity Cultivation in Acid Medium to Improve Bacterial Survival of Freeze-Drying

**DOI:** 10.17113/ftb.59.04.21.7076

**Published:** 2021-12

**Authors:** Aurore Bodzen, Audrey Jossier, Sébastien Dupont, Pierre-Yves Mousset, Laurent Beney, Sophie Lafay, Patrick Gervais

**Affiliations:** 1 University of Burgundy, AgroSup Dijon, PAM UMR A 02.102, 21000 Dijon, France; 2 Indigo Therapeutics, 5 rue Salneuve, 75017 Paris, France

**Keywords:** freeze-drying, viability, membrane fluidity, acid prestress, *Lactococcus lactis*

## Abstract

**Research background:**

Freeze-drying is the most widely used dehydration process in the food industry for the stabilization of bacteria. Studies have shown the effectiveness of an acid prestress in increasing the resistance of lactic acid bacteria to freeze-drying. Adaptation of bacteria to an acid stress is based on maintaining the properties of the plasma membrane. Indeed, the fatty acid composition of the membrane of lactic acid bacteria is often changed after an acid prestress. However, few studies have measured membrane fluidity after an acid stress during lactic acid bacterial strain cultivation.

**Experimental approach:**

In order to use two pH profiles, the strains *Lactococcus lactis* NCDO 712 and NZ9000 were cultivated in two media, without any pH control. The two pH profiles obtained were representative of the initial medium composition, medium buffering properties and strain metabolism. Absorbance at 600 nm and pH were measured during bacterial cultivation. Then, the two strains were freeze-dried and their survival rates determined. Membrane fluidity was evaluated by fluorescence anisotropy measurements using a spectrofluorometer.

**Results and conclusions:**

Cultivation under more acidic conditions significantly increased the survival during freeze-drying (p<0.05, ANOVA) of both strains. Moreover, in both strains of *L. lactis*, a more acidic condition during cultivation significantly increased membrane fluidity (p<0.05, ANOVA). Our results revealed that cultivation under such conditions, fluidifies the membrane and allows a better survival during freeze-drying of the two *L. lactis* strains. A more fluid membrane can facilitate membrane deformation and lateral reorganization of membrane components, critical for the maintenance of cellular integrity during dehydration and rehydration.

**Novelty and scientific contribution:**

A better understanding of the involvement of membrane properties, especially of membrane fluidity, in bacterial resistance to dehydration is provided in this study.

## INTRODUCTION

Freeze-drying is a dehydration process that stabilizes bacteria in order to preserve their long-term viability until they are used. This process is frequently employed in the food industry for the production of lactic acid bacteria used as starters or probiotics in dehydrated form. It has been widely demonstrated that the technological stresses induced by freeze-drying can cause cell damage and, in some cases, cell death. Cellular dehydration, caused in large part by osmotic stress due to the addition of protective solutes and by the freezing itself, is the source of mechanical and structural stresses on cells and cellular components. During severe dehydration, permeabilization of the plasma membrane can be induced and is often reported to be the cause of cell death (*1*). The other critical step in freeze-drying is the rehydration step, which precedes the use of the bacteria and influences both bacterial survival and functionality (*2*). The influence of different rehydration parameters on the viability and functionality of bacteria has been discussed in recent studies, in particular the influence of the kinetics (*3*, *4*) and the presence of oxygen (*5*).

The strategies used to maximize the survival of bacteria during freeze-drying and rehydration mainly include the addition of lyoprotectants (*2*), the application of prestresses which prepare the bacteria, and the optimization of conventional operating parameters of the process (time, pressure and temperature ranges). The exposure of bacteria to moderate stresses during or after cultivation (thermal, osmotic, acid, *etc*.) has also been described as an effective strategy to increase bacterial viability. More specifically, some studies have shown that an acid prestress during cultivation has increased the survival of *Lactobacillus* strains during freezing or freeze-drying (*6*-*9*).

The adaptation of bacteria to an acid stress is based on cell homeostasis (principally *via* an overproduction of adenosine triphosphate (ATP)) and maintaining the properties of the plasma membrane. To maintain cell homeostasis during acid stress, the ATP produced during glycolysis can be redirected to increase the activity of the F-ATPase pump (proton expulsion from the intracellular medium) (*10*). The cells can also modify the pyruvate pathways and thus produce less lactate (*11*). Finally, the adaptation of bacteria to acid stress also involves the modification of the protein pool by the production of chaperone proteins or proteins involved in glycolysis (*12*). To maintain the structural and functional integrity of the plasma membrane, the cell can synthesize *de novo* fatty acids by redirecting the acetyl coenzyme A (acetyl-CoA) produced during glycolysis or modify the fatty acids already present in the membrane. Studies have shown that after an acid stress, the fatty acid composition of lactic acid bacterium membrane is often changed (*6*-*8*, *13*-*15*). An increase of the unsaturated fatty acid (UFA) to saturated fatty acid (SFA) ratio and of cyclopropane fatty acid (CFA) content were reported in these studies. Indeed, modulating the amount of UFAs or SFAs, the amount of CFAs or even the length of the fatty acid chains can thus influence membrane fluidity and bacterial resistance to mechanical stress caused by freeze-drying (*16*, *17*). However, few studies have measured membrane fluidity after an acid stress caused during cultivation of lactic acid bacterial strains. Wang *et al.* (*9*) found that a lower pH during cultivation of a *Lactobacillus acidophilus* strain reduced the loss of acidification activity after freezing at −20 °C and storage for 3 months at −20 °C. The ratio of UFA/SFA and the amount of CFAs were higher after an acid stress. In addition, higher membrane fluidity after cultivation at acidic pH was measured. Nevertheless, the influence of an acid prestress during cultivation on the survival of *Lactococcus* spp. strains during freeze-drying has not been studied yet.

Therefore, the aim of our study is to compare the membrane fluidity and the freeze-drying survival of two strains of *Lactococcus lactis* after cultivation under different acidic conditions.

## MATERIALS AND METHODS

### Bacterial strains and stock solutions

The strain *L. lactis* NCDO 712, a starter used in the manufacture of cheese, was first selected for this study. This strain has 6 plasmids including the plasmid pLP 712 with the genes coding for the transport and metabolism of lactose (*18*). The *L. lactis* NZ9000 strain, which is derived from the *L. lactis* NCDO 712 strain but devoid of plasmids, was also selected for this study. Mutations and the insertion of the regulatory genes *nisR* and *nisK* into the *pepN* and *napC* for the NICE system (gene expression system regulated by nisin) in the *L. lactis* NZ9000 strain created differences compared to the *L. lactis* NCDO 712 strain, in particular with regard to the catabolism of sugars. Based on this assertion and due to the predominant role of ATP in the resistance to acid stress by increasing the activity of the F-ATPase pump, a greater sensitivity of the *L. lactis* NZ9000 strain to acid stress was hypothesized.

The *Lactococcus lactis* NCDO 712 and *Lactococcus lactis* NZ9000 strains were cultured in gM17 medium (M17 broth supplemented with 0.5% glucose) at 30 °C for approx. 16 h. The M17 medium and glucose (both from Sigma-Aldrich, Merck, Darmstadt, Germany) were added after sterilization of the medium at 121 °C for 20 min. The pH of the gM17 medium was adjusted before autoclaving to pH=7.1±0.2 at 25 °C with 1 M sterile NaOH. The cultures were then diluted to 20% (by volume) with sterile glycerol (Honeywell, Morris Plains, NJ, USA), then aliquoted in 1 mL cryotubes and stored at −80 °C.

### Culture conditions

In order to use two pH profiles, the two strains were cultivated in two media commonly used for lactic acid bacterial biomass production (*19*, *20*): MRS medium (*Lactobacillus* broth acc. to De Man, Rogosa and Sharpe, Sigma-Aldrich, Merck) and gM17 medium, without any pH control. As the other culture parameters were controlled (temperature, mixing, *etc*.), the pH profile evolution was characterized and used to assess the fermentation. Indeed, these pH profiles were representative of initial medium composition, buffering properties of the media, and strain metabolism.

From the cryotubes, strains were inoculated by seeding 100 μL of culture on Petri dishes containing gM17 agar medium supplemented with 15 g/L VWR agar (Leuven, Belgium). After 24 h at 30 °C, colonies were inoculated in 10 mL gM17 medium (precultures). Finally, the gM17 and MRS media were inoculated at 1% (by volume) with the precultures and then incubated at 30 and 37 °C respectively for 24 h. The MRS medium was sterilized at 121 °C for 20 min after the addition of 0.1% (by volume) Tween 80 (Sigma-Aldrich, Merck). The pH was adjusted before autoclaving to pH=6.2±0.2 at 25 °C with 1 M sterile NaOH.

### Monitoring of the pH and absorbance during cultivation

In this study, cultivation was carried out without pH regulation and acidic pH was achieved by lactic acid production. The optimum pH for the cultivation of *Lactococcus* strains is between pH=6.3 and 6.9 (*21*). The initial pH of M17 medium was pH=7.1±0.2 since it contained a buffer solution (sodium glycerophosphate). The pH of the M17 medium was not regulated, but its acidification was increasing with the production of lactic acid. As the initial pH of the MRS medium was lower (pH=6.2±0.2) and as this medium did not contain a buffer solution, a more acidic pH was observed during the cultivation of the two strains than in M17 medium.

The pH of the two strains and the two culture media was measured every hour with a pH meter HI1053B equipped with a combined pH probe (Hanna Instruments, Tanneries, France), previously calibrated using buffer solutions (pH=4 and 7).

Absorbance of the two strains and the two culture media was measured every two hours at 600 nm, using a spectrophotometer Genesys 20 (Thermo Fisher Scientific, Waltham, MA, USA).

### Freeze-drying

After cultivation in these two media, the two strains of *L. lactis* were then freeze-dried and their survival rates were determined. The produced biomass was harvested in stationary phase and the number of cells per millilitre was estimated and expressed in CFU/mL. It was then centrifuged (5810 R; Eppendorf, Sigma-Aldrich, Merck) at 4000×*g* for 10 min and the pellets were suspended in 5% (*m*/*V*) sucrose (Sigma-Aldrich, Merck) in phosphate-buffered saline (PBS, Sigma-Aldrich, Merck). Sucrose was added to multiply by 10 the number of cells produced per mL. A volume of 1 mL of this mixture was transferred to vials (5-mL amber glass vials), then frozen at −80 °C (−2 °C/min) before being freeze-dried for 24 h in a FreeZone 18-litre console freeze dry system with stoppering tray dryer, purge valve and PTFE-coated collector (Labconco, Kansas City, MO, USA). Sublimation was carried out by maintaining the samples at −40 °C for 2 h (condenser temperature at −55 °C and chamber pressure at 10 Pa), then increasing the chamber temperature to 0 °C at a heating rate of 0.04 °C/min. After about 17 h, the temperature of the chamber was increased to 25 °C at a heating rate of 0.08 °C/min to carry out the secondary desorption. The samples were then sealed under vacuum before rehydration with 1 mL of gM17 medium at 30 °C in order to determine the final biomass. The final biomass after freeze-drying was expressed in CFU/mL. The survival rate during freeze-drying (%) was expressed by calculating the ratio between the viable biomass after freeze-drying (CFU/mL after freeze-drying) and the biomass produced (CFU/mL before freeze-drying).

### Membrane fluidity

The membrane fluidity of the bacterial cells was evaluated by fluorescence anisotropy using a spectrofluorometer (Fluorolog®-3, HORIBA, Longjumeau, France) in T format (*22*-*24*). As before, the cells were harvested in stationary phase after culture in gM17 or MRS media and then washed twice in 15 mM TRIS-HCl buffer (Trizma® hydrochloride, pH=7; Sigma-Aldrich, Merck) and centrifuged (4000×*g*, 10 min, 25 °C). The cells were then resuspended in the same buffer to reach an absorbance of 0.250 at 600 nm (*i.e.* 2.5·10^8^ CFU/mL). The hydrophobic fluorescent probe 1,6-diphenyl-1,3,5-hexatriene (DPH; Invitrogen Molecular Probes, Sigma-Aldrich, Merck) was used (*23*) and a 10 mM stock solution in tetrahydrofuran was prepared. A volume of 3 mL of samples was placed in a quartz cell and 2 μL of the stock solution of DPH (*c*=6.7 μM) were added using a Hamilton glass syringe. After incubating this suspension at 30 °C for 5 min, the measurements were carried out every 5 °C during the cooling step from 30 °C (optimal temperature for the growth of the strains) to 5 °C (temperature allowing the medium to remain liquid for the measurement by fluorescence anisotropy). Two anisotropy measurements were performed for each temperature condition. The excitation and emission wavelengths were (360±5) and (430±5) nm, respectively (*25*). According to Lakowicz (*26*), the fluorescence anisotropy (*r*) is the ratio of the difference in the intensity of the emitted parallel and perpendicular polarized light (I_||_−I_⊥_) to the total intensity of polarized light emitted by the sample (I_||_+2I_⊥_).

### Statistical analysis

An analysis of variance (p<0.05, ANOVA) was carried out to determine whether differences in biomass production, freeze-drying survival and biomass present after freeze-drying were significant between the culture media. The experiments were carried out in triplicates (three independent cultures). The results are presented as the mean values with standard deviations (*N*=3). The R Software v. 3.3.2 was used to statistically analyse the data (*27*).

## RESULTS AND DISCUSSION

### pH profiles obtained during bacterial cultivation in the two media

#### pH profiles

**Fig. 1:**
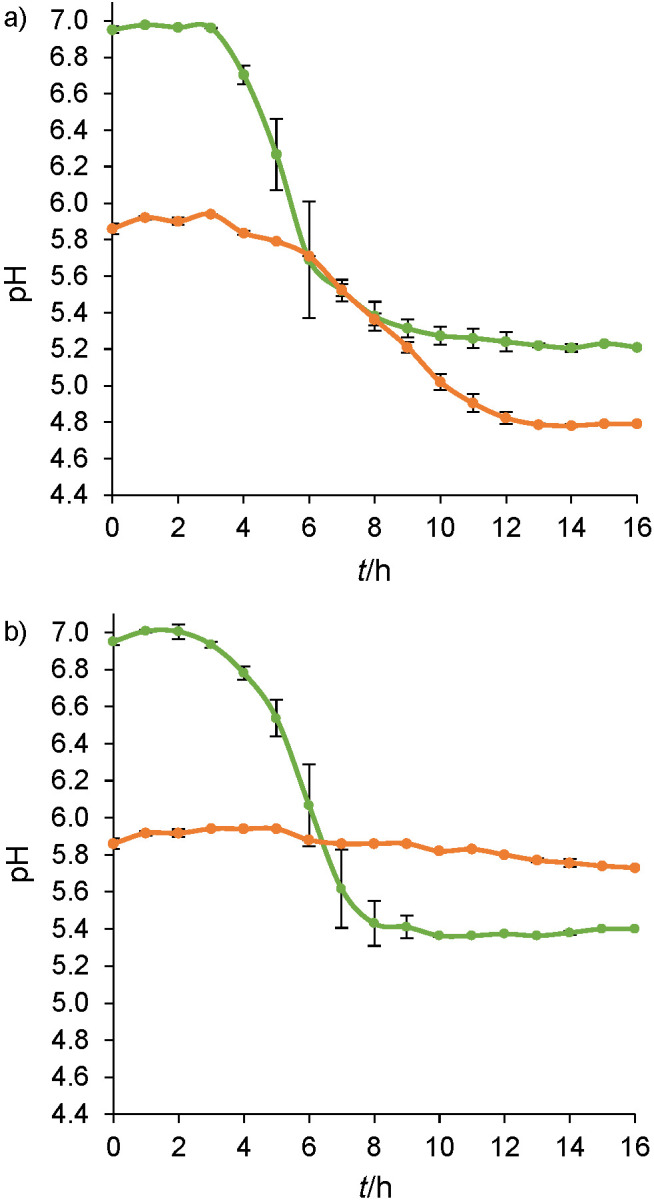
The pH of: a) *Lactococcus lactis* NCDO 712 and b) *Lactococcus lactis* NZ9000 during 16 h of cultivation in gM17 (green) and MRS (orange) media. Values represent mean±standard deviation obtained from independent triplicate measurements

In order to define the two pH profiles during the cultivation of the two strains of *L. lactis* in the gM17 and MRS media, the pH and absorbance were measured. Fig. 1 presents the results of monitoring the pH during cultivation of *L. lactis* NCDO 712 (Fig. 1a) and *L. lactis* NZ9000 (Fig. 1b) strains as a function of the culture medium (gM17 or MRS).

As shown in Fig. 1, the pH of the gM17 medium after sterilization (and therefore at the start of the cultivation) was close (pH=6.95±0.02) to that before sterilization. The pH of the MRS medium decreased after sterilization. Before sterilization, the pH was 6.2 and at the start of cultivation it was 5.9±0.3.

During cultivation of *L. lactis* NCDO 712 strain, pH=6.95 was stable for 4 h in gM17 medium. Then, the pH decreased very quickly until reaching minimum values close to 5 (pH=5.15±0.03) from 9 h of cultivation and thereafter remained stable until harvest. The pH curve obtained during cultivation of *L. lactis* NZ9000 strain in gM17 medium was close to that of *L. lactis* NCDO 712. Concerning the culture of the two strains in the MRS medium, the pH curves were different from those obtained in gM17 medium and different between the two strains. During cultivation of *L. lactis* NCDO 712 strain, the pH=5.9 was stable for 3 h, then it decreased rapidly until reaching values close to 5 (pH=4.66±0.01) after 12 h of culture, thereafter remaining stable until harvest. For the *L. lactis* NZ9000 strain, the pH=5.9 was stable for 10 h and then decreased very slowly, reaching pH=5.69 at 16 h of culture. Unlike *L. lactis* NCDO 712 strain, the pH at harvest of *L. lactis* NZ9000 strain in the MRS medium was higher than in the gM17 medium. Table 1 presents the time of cultivation in different pH ranges (optimal, acidic and very acidic pH according to Hutkins and Nannen (*21*)) for each strain and each culture medium (total cultivation time of 16 h).

**Table 1:** Time of the cultivation in each medium during which *Lactococcus lactis* NCDO 712 and *Lactococcus lactis* NZ9000 strains were at optimal pH=6.3–7, at acidic pH=5.3–6.3 that allowed growth and at pH below which growth is limited (pH<5.3)

**pH**	*t*(cultivation)/h			
gM17		MRS	
NCDO 712	NZ9000	NCDO 712	NZ9000
**6.3–7**	5	5	0	0
**5.3–6.3**	4	11	9	16
**<5.3**	7	0	7	0

Both strains of *L. lactis* spent the same time at optimal pH (5 h at pH=6.3-7) during cultivation in gM17 medium. During cultivation of the strains in the MRS medium, the initial pH was 5.9, and thus the start of the growth of the two strains did not take place in an optimal pH range.

In the case of the *L. lactis* NCDO 712 strain, the pH of the medium reached values of pH<5.3 for 7 h at the end of cultivation in both media. Unlike *L. lactis* NCDO 712 strain, the pH of both media during cultivation of *L. lactis* NZ9000 strain never reached pH<5.3.

As expected, two pH profiles were observed after cultivation of *L. lactis* NZ9000 and *L. lactis* NCDO 712 in the two media. Both strains were subjected to acidic pH during their cultivation in gM17 and MRS media. However, a more acidic pH was obtained for both strains in the MRS medium than in the gM17 medium.

#### Impact of pH profiles on bacterial growth

**Fig. 2:**
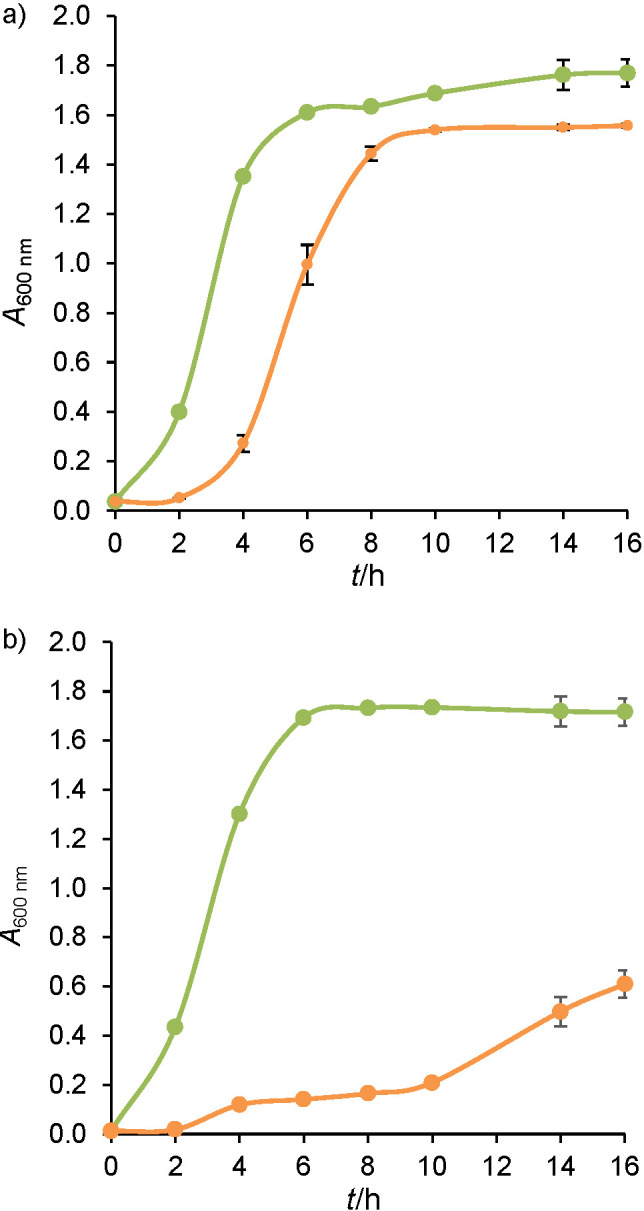
Absorbance at 600 nm of: a) *Lactococcus lactis* NCDO 712 and b) *Lactococcus lactis* NZ9000 during 16 h of cultivation in gM17 (green) and MRS (orange) media. Values represent mean±standard deviation obtained from independent triplicate measurements

Fig. 2 shows the monitoring of growth by measuring the absorbance at 600 nm for *L. lactis* NCDO 712 strain (Fig. 2a) and for *L. lactis* NZ9000 strain (Fig. 2b) in different culture media (gM17 or MRS).

The same growth phases were observed of both *L. lactis* NCDO 712 and *L. lactis* NZ9000 during cultivation in gM17 medium. Regarding the monitoring of growth during the cultivation of *L. lactis* NCDO 712 in MRS medium, a latency phase of 2 h was observed. The exponential phase was therefore observed up to 8 h of culture (maximum specific speed of growth being similar between the two media). The absorbance reached in the stationary phase was higher if the cultivation took place in gM17 medium, which means that the biomass content produced in this medium should be higher than in MRS medium. The presence of a latency phase during cultivation in the MRS medium was probably due to the change of cultivation medium between the precultivation and the cultivation and to the time of bacterial adaptation to this new medium.

The growth curve of *L. lactis* NZ9000 strain in the MRS medium is different from that obtained in gM17 and from that obtained for *L. lactis* NCDO 712. A latency phase of 2 h was observed, followed by a first phase of growth acceleration (up to 10 h of cultivation) then a second, until the stationary growth phase was reached after 20 h of cultivation (results not shown). The bacterial biomass (after 16 h of cultivation) was therefore harvested when the bacterial cells were in the growth phase. The biomass content during the harvest in the MRS medium should therefore be much lower than that obtained in the gM17 medium. The initial non-optimal pH of the gM17 medium caused a strong acid stress for this strain, which resulted in weak growth and a longer adaptation to this culture medium than that observed in the *L. lactis* NCDO 712 strain.

The growth curves obtained in this study as well as the pH variations during cultivation were very consistent with previous studies from the literature (*28*, *29*). Liu *et al*. (*28*) cultivated a strain of *Lactococcus lactis* in MRS and M17 media without pH regulation. After a latency phase of 3 h, the stationary growth phase was reached after 10 h in the M17 medium (an exponential growth phase of 7 h). Likewise, the pH varied from pH=6.8 to 5 between the start and the end of growth. Concerning the culture grown in the MRS medium, a lower absorbance at 600 nm (difference of 0.3) as well as a lower pH (pH=4.7) were obtained at the end of the cultivation than in M17 medium (*28*). In another study, a strain of *Lactococcus lactis* was grown in a modified MRS medium without pH control. As observed in our study, the stationary phase was reached after 8 h and the pH varied during growth from pH=6.5 to 4.4 (*29*).

A comparison of Fig. 1 and Fig. 2 shows that the pH of the medium varied as a function of the growth of the strains. Indeed, the acidification of the culture medium was correlated with the onset of bacterial growth and increased proportionally to it. The slowing down and then the stop of growth also seems to correlate with the stabilization of the pH at the minimum observed values. As widely documented, during the production of lactic acid bacterial biomass, acidic pH is the main growth inhibiting factor (*21*). In lactic acid bacteria such as lactococci, the production of ATP for cell multiplication is accompanied by the production of lactic acid. The secretion of lactic acid acidifies the external environment and is responsible for the acidification of the culture media, so the bacterial cells are no longer able to maintain an internal pH suitable for growth (*21*). The resulting internal acidification can reduce the activity of certain enzymes and cause damage to proteins and DNA. This acid stress can therefore result in slower growth and cell death. This close relationship between the pH and growth exists whatever the medium considered. The pH of a culture medium is therefore a predictive variable of the biomass production and is affected by the composition of the culture medium.

Moreover, as the growth of *L. lactis* NZ9000 was much more affected than the growth of *L. lactis* NCDO 712 in MRS medium, it would seem that this strain is more sensitive to acidic pH during cultivation. Our hypothesis concerning the difference in sensitivity to acid stress between the two strains is thus confirmed. This difference could be explained by comparing the genomes of these two strains (*18*, *30*). The analysis of the *L. lactis* NZ9000 genome revealed several differences, mostly point mutations, with the genome of *Lactococcus lactis* MG1363 (with a derivative of strain *L. lactis* NCDO 712). Comparative transcriptomic studies revealed that some of these mutations were predicted to affect the function of proteins, in particular glyceraldehyde-3-phosphate dehydrogenase (*gapB*), proteins involved in the transport and metabolism of amino acids (ex: *aroD, gltX*) and the chaperone protein GroEL (*groEL*).

The role of proteins included in the metabolism of amino acids encoded by the *gltX* and *aroD* genes in the resistance and adaptation to acid stress, *via* their contribution to the synthesis of ATP, can therefore be presumed. Among the genes involved in the acid stress response of *L. lactis*, *groEL* has also been identified (*31*). Glyceraldehyde-3-phosphate dehydrogenase (GAPDH) has been recognized as playing an important role in bacterial resistance to acid stress, providing the ATP necessary to maintain homeostasis (*32*). Consequently, mutations affecting the *gltX, aroD, groEL* and *gapB* genes in the *L. lactis* NZ9000 strain may explain its increased sensitivity to acidic conditions during cultivation compared to the *L. lactis* NCDO712 strain.

### Influence of pH profiles on the survival of strains during freeze-drying

**Fig. 3:**
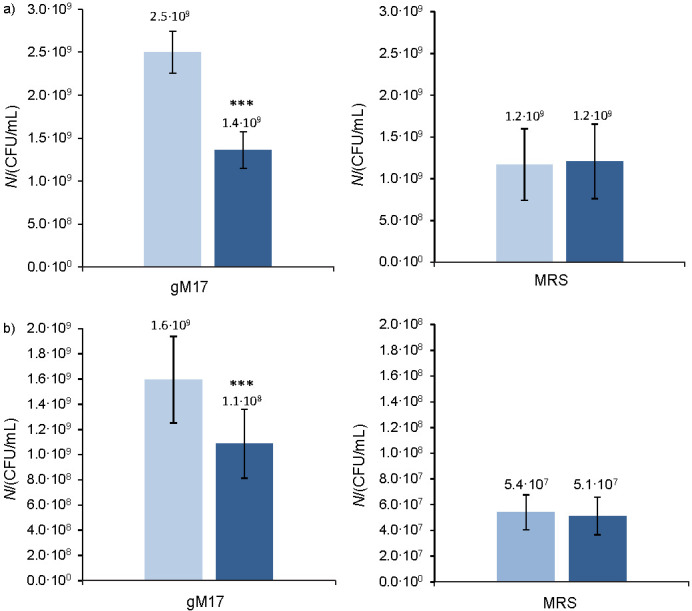
Cultivable biomass (light blue) and final cultivable biomass after freeze-drying (dark blue) produced by: a) *Lactococcus lactis* NCDO 712 and b) *Lactococcus lactis* NZ9000 as a function of the culture medium (gM17 and MRS). FD=freeze-drying. The asterisks indicate a significant difference between the produced biomass and final biomass after freeze-drying (*p<0.05, **p<0.01, ***p<0.001, ANOVA). Values represent mean±standard deviation obtained from independent triplicate measurements

Fig. 3 shows the cultivable biomass (CFU/mL) and the final cultivable biomass after freeze-drying (CFU/mL) produced by *L. lactis* NCDO 712 (Fig. 3a) and *L. lactis* NZ9000 (Fig. 3b) strains in the two culture media (gM17 and MRS).

In the gM17 medium, *L. lactis* NCDO 712 strain (Fig. 3a) produced significantly higher (p<0.05, ANOVA) amount of biomass than *L. lactis* NZ9000 strain (Fig. 3b). The biomass (CFU/mL) produced by both strains after 16 h of cultivation in the MRS medium was significantly lower (p<0.05, ANOVA) than that after 16 h in the gM17 medium (Fig. 3), a finding that is in agreement with the results obtained by absorbance measurement (Fig. 2). For the *L. lactis* NCDO 712 strain, the final biomass amount after freeze-drying was not significantly different (p>0.05, ANOVA) between the two culture media (Fig. 3a). For the *L. lactis* NZ9000 strain (Fig. 3b), the final biomass amount after freeze-drying was significantly lower when it was cultivated in MRS medium (5.1·10^7^ *vs* 1.1·10^9^ CFU/mL in gM17 medium).

**Fig. 4:**
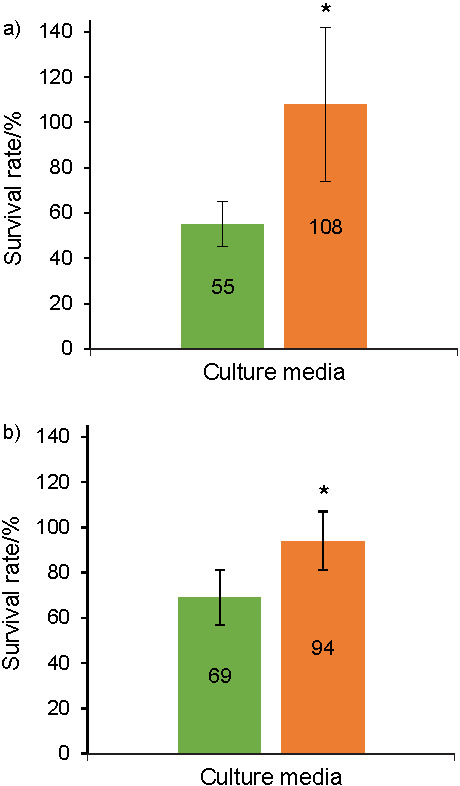
Freeze-drying survival rate of: a) *Lactococcus lactis* NCDO 712 and b) *Lactococcus lactis* NZ9000 as a function of the culture medium (gM17 in green and MRS in orange). The asterisks indicate a significant difference between the two conditions (*p<0.05, **p<0.01, ***p<0.001, ANOVA). Values represent mean±standard deviation obtained from independent triplicate measurements

Fig. 4 shows the freeze-drying survival rate (%) of *L. lactis* NCDO 712 (Fig. 4a) and *L. lactis* NZ9000 (Fig. 4b) depending on the culture medium (gM17 or MRS).

Regarding the survival rates during freeze-drying (Fig. 4), they were significantly higher (p<0.05, ANOVA) in the MRS than in the gM17 medium ((108±4) and (55±10) %, respectively, for *L. lactis* NCDO 712 and (94±13) and (69±12) %, respectively, for *L lactis* NZ9000). The survival of both strains during freeze-drying was significantly higher (p<0.05, ANOVA) after cultivation in the MRS medium. Our hypothesis was therefore that cultivation in the MRS medium, *i.e*. under more acidic conditions than in the gM17 medium, was responsible for increasing the survival of these *L. lactis* strains during freeze-drying. The components of the MRS and gM17 media may have played a role in the freeze-drying survival of the strains. However, some studies on lactobacilli have shown that an acid stress during cultivation increased their survival during freezing or freeze-drying (*6*-*9*). Nevertheless, the influence of medium components on the survival of these two strains during freeze-drying could be investigated in future experiments.

Although the production of biomass by *L. lactis* NCDO 712 strain was significantly lower in MRS medium than in gM17 medium (1.2·10^9^ *vs* 2.5·10^9^ CFU/mL), the survival rate during freeze-drying was significantly higher (108 *vs* 55%), and the cultivable biomass after freeze-drying therefore remains high (1.2·10^9^ *vs* 1.4·10^9^ CFU/mL). The importance of cultivation conditions for this strain, not maximizing the production of biomass but maximizing the survival during freeze-drying, is therefore verified. Shao *et al*. (*7*) made the same observation; even at the end of fermentation, the biomass of *Lactobacillus delbrueckii* strain was lower at pH=5.1 than at pH=5.7 and freeze-drying survival was higher, which resulted in a higher viable biomass after freeze-drying. In the case of *L. lactis* NZ9000, even if cultivating conditions made it possible to obtain a high survival during freeze-drying (MRS: 94% *vs* gM17: 69%), the amount of biomass produced in the MRS medium was nonetheless very low at the end of cultivation (MRS: 5.4·10^7^ CFU/mL *vs* gM17: 1.6·10^9^ CFU/mL). It thus seems that the cultivating conditions for this strain were too stressful (initial pH of the MRS medium<initial pH of the gM17 medium) for the production of biomass. Optimization of cultivating conditions encouraging good production of biomass and survival during freeze-drying would therefore be an essential step in any industrial use of this strain. Several strategies may be developed: cultivation in a medium with a non-optimal but less acidic pH (5.9–6.3 with or without pH regulation), an acid shock at the end of cultivation, or cultivation until the end of the exponential phase conducted under optimal pH conditions, followed by acid stress for a few hours.

In order to understand the origin of the improved survival of the strains during freeze-drying following acidic pH during cultivation, the structural and functional properties of the bacterial plasma membrane were examined. Although acid stress can also have consequences on cellular homeostasis and in particular on the increased production of ATP, the implication of the plasma membrane in the resistance to freeze-drying seems a stronger hypothesis. Indeed, the plasma membrane is the main target of the cellular damage that occurs during a dehydration/rehydration cycle. The organization of membrane phospholipids in the liquid-crystalline phase or in the gel phase varies depending on the temperature and degree of hydration. This is why, during dehydration, the withdrawal of water leads to the destabilization of the membrane structure and a transition from the liquid-crystalline phase to a gel phase, resulting in membrane rigidification. The coexistence of the different lipid phases has been identified as responsible for a reduction in the resistance of the membrane to shear forces and therefore for the possibility of cell permeabilization during dehydration (*16*, *23*). In addition, the cell shrinkage caused by dehydration leads to an increase in the surface area/volume ratio of the cells. Due to the low lateral compressibility of the plasma membrane, the membrane deforms and invaginations are formed, leading to the formation of intracellular vesicles responsible for a loss of membrane surface (*33*). This loss of surface is at the origin of a permeabilization of the membrane during the rehydration phase (*34*, *35*). The permeabilization of the membrane during rehydration explains cell death. The deformation of the membrane as well as its mechanical resistance therefore depend on its properties and in particular on its fluidity (*16*, *36*-*38*). A study of membrane fluidity was therefore undertaken.

### Modulation of membrane fluidity after cultivation at two pH values and impact on survival during freeze-drying

#### Estimation of the membrane fluidity of the two strains

**Fig. 5:**
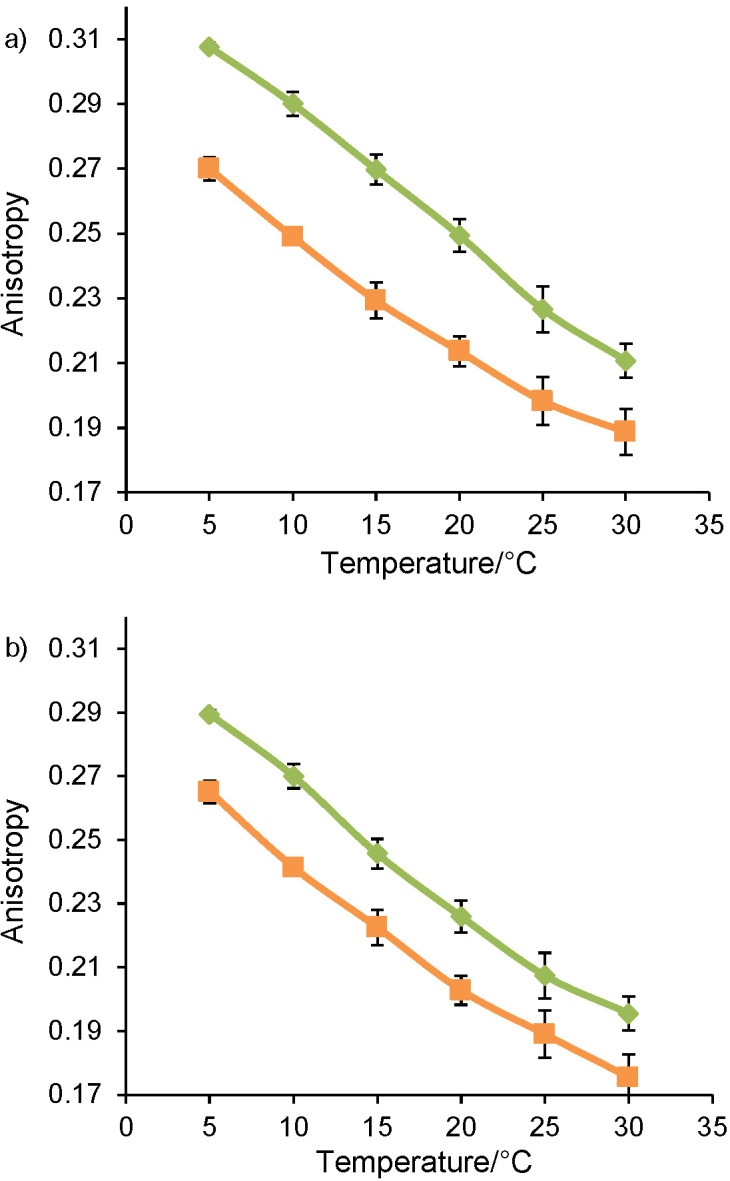
Anisotropy values of: a) *Lactococcus lactis* NCDO 712 and b) *Lactococcus lactis* NZ9000 as a function of the culture medium (gM17 in green and MRS in orange) and temperature. Values represent mean±standard deviation obtained from two independent experiments

Fig. 5 shows the fluorescence anisotropy values influenced by temperature and culture medium for *L. lactis* NCDO 712 (Fig. 5a) and *L. lactis* NZ9000 (Fig. 5b) strains.

The anisotropy values vary with temperature for both strains, whatever the culture medium. The lower the temperature, the higher the anisotropy value. The anisotropy values are inversely related to membrane fluidity; the plasma membrane becomes more rigid with the lowering of the temperature.

#### Influence of pH values on membrane fluidity during cultivation

The culture medium has a significant influence (p<0.05, ANOVA) on the anisotropy values for both strains, and these values are lower for bacterial strains cultivated in MRS medium. Hence, membrane fluidity of the two *L. lactis* strains was higher after cultivation in the MRS medium than in the gM17 medium. Therefore, a more acidic pH during cultivation of the two *L. lactis* strains increased bacterial membrane fluidity.

Moreover, during cultivation of *L. lactis* NZ9000, the pH of the MRS medium was higher than the pH of the gM17 medium at the harvest time. However, the membrane fluidity of the strain was higher after cultivation in the MRS than in the gM17 medium. These results reveal that the membrane fluidity of *L. lactis* NZ9000 did not depend solely on the final pH but rather resulted from the acidic pH during entire cultivation.

#### Influence of membrane fluidity on freeze-drying survival and role of pH values

**Fig. 6:**
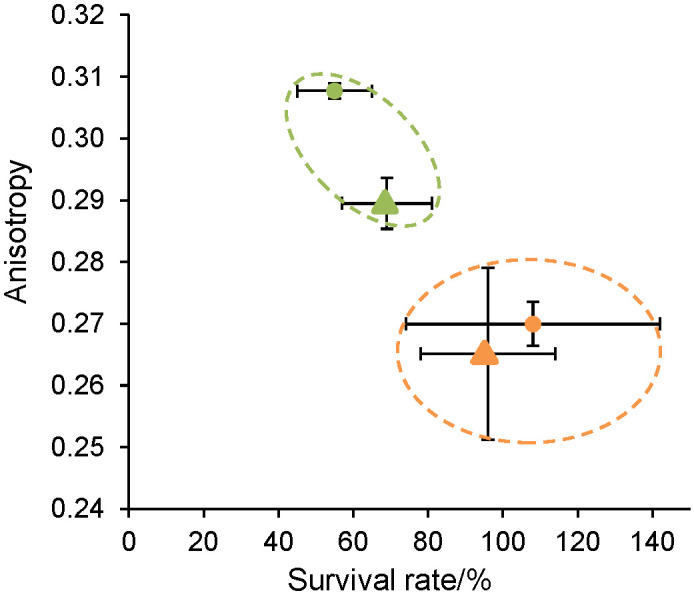
Fluorescence anisotropy at 5 °C of *Lactococcus lactis* NCDO 712 (circles) and of *Lactococcus lactis* NZ9000 (triangles) according to their survival rate during freeze-drying and to the culture medium (gM17 in green and MRS in orange)

Fig. 6 presents the anisotropy value obtained under each parameter (membrane fluidity and pH) as a function of bacterial survival rate during freeze-drying.

A link between the anisotropy values and the survival during freeze-drying is observed: the lower the anisotropy value (*i.e*. the more fluid the membrane), the higher the freeze-drying survival rate. There even seems to be an anisotropy value threshold (~0.28) below which freeze-drying survival is greater than 80%. Meneghel *et al*. (*39*, *40*) have also demonstrated that a higher membrane fluidity allowed better survival of *Lactobacillus delbrueckii* strains during osmotic and cold stress, thus a better cryotolerance. In our study, modification of membrane fluidity was induced by means of an acid pre-stress. Cultivation of the *L. lactis* strains in the MRS medium, *i.e*. under a more acidic condition than in the gM17 medium, resulted in higher bacterial membrane fluidity. These results were in agreement with those of Wang *et al*. (*9*) with a *Lactobacillus acidophilus* strain. An acid stress during cultivation increased membrane fluidity, which resulted in an improved cryotolerance of the strain. Other studies have also shown that modulation of fatty acid composition after an acid prestress during cultivation increased *Oenococcus oeni* (*14*, *15*) and *Lactobacillus* spp. strain (*6*-*8*) survival during freezing or freeze-drying.

However, on the contrary, some studies have shown a membrane rigidification and a decrease in the UFA/SFA ratio after an acid stress (*41*, *42*). In these studies, acid shock and strong acid stress were observed. The decreased membrane fluidity therefore allowed bacteria to counter the influx of protons and to better resist acid stress (*41*). Thus, if an acid prestress is induced in order to increase the survival of bacterial strains to dehydration, it should not be too strong or too long to avoid the corresponding membrane rigidification. Indeed, a strong membrane rigidification can increase its rupture during the mechanical stress caused by dehydration (*16*, *43*, *44*). Therefore, the exposure time/pH value must be carefully chosen to achieve an acid prestress during cultivation. To *et al*. (*45*) showed a decrease of UFA/SFA ratio and an increase of CFAs after cultivation of a *Lactococcus lactis* strain at pH=5, but without a significant difference in membrane fluidity compared to a cultivation at pH=7. Nevertheless, the authors have suggested that *L. lactis* adaptation to stresses depends on membrane fluidity modulation by a balance between the amounts of palmitoleic, *cis*-vaccenic and lactobacillic acids. Therefore, in further experiments, it would be interesting to determine the composition of membrane fatty acids, and particularly those three previously mentioned, after cultivation at the two pH values used in this study. Some other studies have observed that membrane rigidification allows better bacterial survival during freezing or long-term storage (*46*, *47*), which is consistent with our study. In these studies, fluorescence anisotropy measurements were performed on cells harvested at different growth phases (from the middle exponential phase to the late stationary phase) and bacterial membrane fluidity was found lower in bacteria harvested in stationary phase than in exponential phase. The authors suggest that this membrane rigidification was correlated with a higher bacterial resistance to freeze-drying and storage. Nevertheless, several other morphological and physiological changes in cells occur between the exponential phase and the stationary phase and could be at the origin of such an increased resistance. Better resistance of bacterial cells harvested in the stationary phase to numerous stresses is generally explained by several factors: the activation of the sigma factor, synthesis of proteins, modification of the cell wall and cell cytoplasmic membrane, modification of the protein/lipid ratio in the membrane, accumulation of compatible solutes, *etc*. (*48*). In our study, cells were always harvested at the same time in stationary phase, which prevents any bias due to the culture growth phase.

The comparison of our results with the literature confirms the main role of acidic pH during cultivation in the survival of bacterial strains and membrane fluidity change during freeze-drying. Higher fluidity allows the maintenance of the structural and dynamic properties of the membrane as well as the functionality of membrane proteins during dehydration and rehydration.

## CONCLUSIONS

Using an acidic medium during cultivation of two strains of *Lactococcus lactis*, NCDO 712 and NZ9000, is an effective strategy to significantly increase their resistance to freeze-drying. Our results show that the freeze-drying resistance of the *L. lactis* strains depends on the maintenance of the structural and mechanical properties of the plasma membrane and in particular on the membrane fluidity. The rigidification of the membrane, a consequence of the withdrawal of water during dehydration, reduces the mechanical resistance of the membrane to shear forces and to variations in cell volumes that take place during a freeze-drying/rehydration cycle. Moreover, our results revealed that using an acidic medium during the cultivation of the two strains of *L. lactis* fluidifies the membrane and allows a better survival of both strains to freeze-drying. Culture conditions must be optimised carefully for each bacterium and each medium to maximize the production of biomass while increasing the bacterial resistance to freeze-drying. For instance, moderate acidic pH may be considered in the case of strains whose cultivation is sensitive to acid stress, such as *L. lactis* NZ9000. Taken together, our results suggest that adapted pH profiles during biomass production can greatly increase the survival of lactic acid bacteria used as starters or probiotics during stabilization process.
